# Effect of Alcohol on the Arteries

**Published:** 1885-06

**Authors:** 


### Effect of Alcohol on Arteries.

Dr. Loomis, of New York, on presenting a case of aneurism to his class made the following pointed statement touching the causative relation of alcohol to this accident: "A man can take two or three glasses of stimulants through the day as he may feel the inclination, and he may continue this habit for perhaps twenty years without any evident harm accruing from it; but, when this man reaches that period of life when the vital powers are on the decline, he suddenly feels himself old before his time, for he has all these years been laying the foundation of a chronic endoarteritis. I believe, gentlemen, that fifty per cent. of all these diseases arise from the use of alcoholic stimulants. The more I see of disease, the more I am convinced that, as a rule, a man is young just in proportion as his arteries are healthy, and old as they are diseased."—*Medical Age.*

OVERWORKED NERVOUS SYSTEMS.—Dr. Edward L. Duer, Philadelphia, Pa., says: "I have used Horsford's Acid Phosphate for years, considering it not only valuable in overworked nervous systems, but alike so in the exhausted condition following protracted fevers."

